# Comparative proteomic analysis of okra (*Abelmoschus esculentus* L*.*) seedlings under salt stress

**DOI:** 10.1186/s12864-019-5737-7

**Published:** 2019-05-16

**Authors:** Yihua Zhan, Qingfei Wu, Yue Chen, Mengling Tang, Chendong Sun, Junwei Sun, Chenliang Yu

**Affiliations:** 1 State Key Laboratory of Plant Physiology and Biochemistry, College of Life Sciences, Zhejiang University, Hangzhou, 310058 China; 2 Institute of Agricultural Equipment, Zhejiang Academy of Agricultural Sciences, Hangzhou, 310021 China; 3 Institute of Horticulture, Zhejiang Academy of Agriculture Science, Hangzhou, 310021 China; 4 The Key Laboratory for Quality Improvement of Agricultural Products of Zhejiang Province, School of Agriculture and Food Science, Zhejiang A&F University, Linan, Hangzhou, 311300 China; 5 College of Modern Science and Technology, China Jiliang University, Hangzhou, 310018 China

**Keywords:** Differential expression protein, Heat shock proteins, Okra, Proteomics, Salt stress, TMT labeling

## Abstract

**Background:**

Salinization seriously threatens land use efficiency and crop yields across the world. Understanding the mechanisms plants use to protect against salt stress will help breeders develop salt-tolerant vegetable crops. Okra (*Abelmoschus esculentus* L.) is an important vegetable crop of the mallow family, which is now cultivated in warm regions worldwide. To understand the effects of salt stress on the protein level of okra, a comparative proteomic analysis of okra seedlings grown in the presence of 0 or 300 mmol L^− 1^ NaCl treatment was performed using an integrated approach of Tandem Mass Tag labeling and LC-MS/MS integrated approach.

**Results:**

A total of 7179 proteins were identified in this study, for which quantitative information was available for 5774 proteins. In the NaCl/control comparison group, there were 317 differentially expressed proteins (DEPs), of which 165 proteins were upregulated and 152 proteins downregulated in the presence of NaCl. Based on the above data, we carried out a systematic bioinformatics analysis of proteins with information, including protein annotation, domain characteristics, functional classification, and pathway enrichment. Enriched gene ontology and Kyoto Encyclopedia of Genes and Genomes pathway analysis showed that the DEPs were most strongly associated with “response to stress” and “protein processing in endoplasmic reticulum”. Furthermore, several heat shock proteins were identified as DEPs.

**Conclusions:**

This information provides a reference direction for further research on the okra proteome in the downstream of the salt stress response, with our data revealing that the responses of okra to salt stress involves by various pathways.

**Electronic supplementary material:**

The online version of this article (10.1186/s12864-019-5737-7) contains supplementary material, which is available to authorized users.

## Background

Soil salinization is one of the major abiotic stresses affecting plant growth and threatening agricultural production, and is a problem that continues to spread worldwide [1] [2]. The increase in salinization leads to an annual global the loss of 10 million hectares of farmland [3] . By 2050, over 50% of the world’s cultivated land is predicted to be salinized [4]. NaCl is the most common salt at present and it has always been the focus of salinity research [5, 6]. High concentrations of NaCl in salinized soil affect plant growth at different physiological levels. It can cause water deficit, ionic toxicity, nutritional imbalance and reactive oxygen species (ROS) production, giving rise to protein and nucleic acid damage, growth and yield decline, and even plant death [7]. Plants have evolved effective strategies to withstand under these various salt-induced stresses. For example, osmotic regulation in the face of salt stress can be achieved by the plant accumulating soluble osmotic protectant substances including proline, polyol betaines, and soluble sugars [8]. Research is providing insights into the molecular and biochemical basis of plant stress tolerance, with the ultimate goal of developing crop cultivars capable of achieving increased yield under salinized conditions.

Okra (*Abelmoschus esculentus* (L.) *Moench*), also known as qiukui, lady’s fingers and quimgombo, is an annual herb and a vital vegetable crop of the mallow family [9]. Okra is grown for its immature pods, which are rich in fiber and vitamins [10]. It is widely cultivated in warm regions around the world [11]. In recent years, many researchers have studied the tolerance of okra to various abiotic stresses. Okra has the ability to tolerate arsenic stress, but cadmium (Cd) accumulation in okra has negative effects on the physiological and biochemical characteristics, growth and development, and yield of okra, meaning that this plant may not be a suitable crop for cultivation in Cd-contaminated soil [12, 13]. Omics technologies are potentially important tools to enhance our understanding of how to improve okra growth and yield under adverse environmental factors [14].

There is limited genome sequence information available on okra. Proteomics analysis is a tool to facilitate the study of global protein expression, and to provide a wealth of information on the role of individual proteins in specific biological processes. There have been many studies on proteomic changes in response to NaCl treatment in plants such as *Arabidopsis* [15], rice [16, 17], barley [18], wheat [19, 20], maize [21], soybean [22], tomato [23], and cucumber [24]. Thirty differentially abundant proteins in response to salinity, which were involved in four types of biological processes in oat leaves, were detected by two dimensional gel electrophoresis (2-DE) and matrix-assisted laser desorption/ionization time-of-flight (MALDI-TOF) mass spectrometry [25]. A total of 128 DEPs were identified from salt-treated cotton (*Gossypium hirsutum* L.) roots by the isobaric tag for relative and absolute quantitation (iTRAQ)-based proteomic technique. Most of DEPs had functions related to stress response and defense [26]. However, there is no proteomic data from okra have been reported to date. Recently, a MS/MS-based tandem mass tags (TMT) label analysis strategy has been developed for large-scale protein quantification [27]. Most studies have focused on salt-induced responses in shoot tissues, because reducing the accumulation of toxic ions in leaves is essential for plant growth and yield [6, 28]. In this study, we employed a TMT label-based quantitative proteomics approach to identify differentially expressed proteins (DEPs) under NaCl treatment. Our comprehensive analysis provides useful information with which to explore the roles of candidates proteins in minimizing the damage caused by salt stress in okra.

## Result

### Quantitative proteomic data analysis

**Fig. 1:**
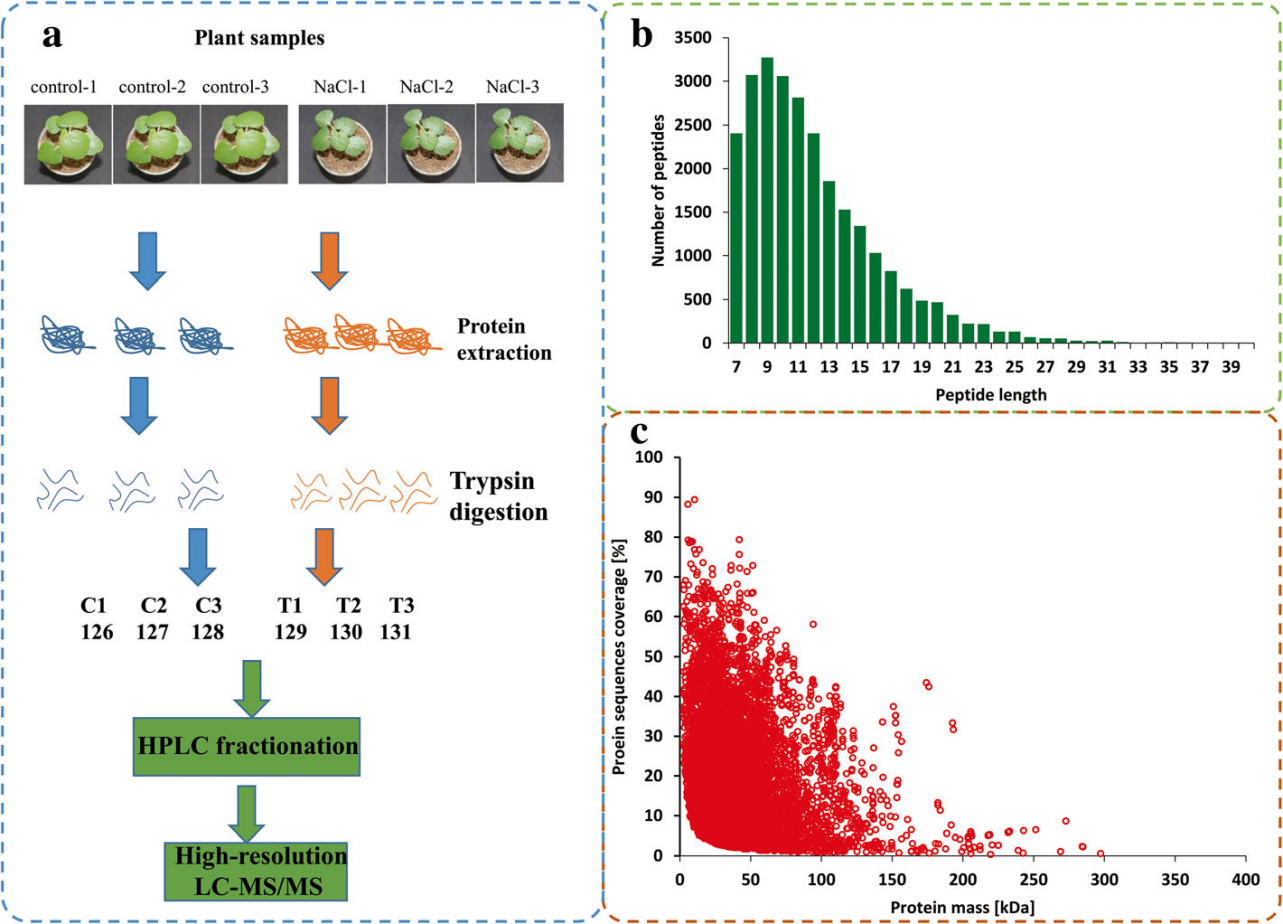
Experimental strategy for quantitative proteome analysis and quality control (QC) validation of MS data. **a** Protein were extracted and trypsin digested in three biological replicates for each sample group. All protein samples were analyzed by HPLC-MS/MS.126–131 label: TMT-126-131 Label Reagent (ThermoFisher Scientific, Shanghai, China). **b** Length distribution of all identified peptides. **c** Mass delta of all identified peptides

Using liquid chromatography–tandem mass spectrometry (LC-MS/MS) and TMT labeling, the proteomic changes of okra seedlings treated with salt or water were analyzed and compared. Our workflow is shown in Fig. 1 a. Pearson’s correlation coefficient between six samples (three replicates × two groups) showed in Additional file 2**:**Figure S1a. Most of the peptides were distributed between eight and 20 amino-acid residues long (Fig. 1 b), a finding which agreed with the typical peptide sizes generated by trypsin digestion, indicating that the sample preparation reached the standard required. The detail information of identified peptides pertinent to detected proteins was listed in Additional file 3**:** Table S2.

**Fig. 2:**
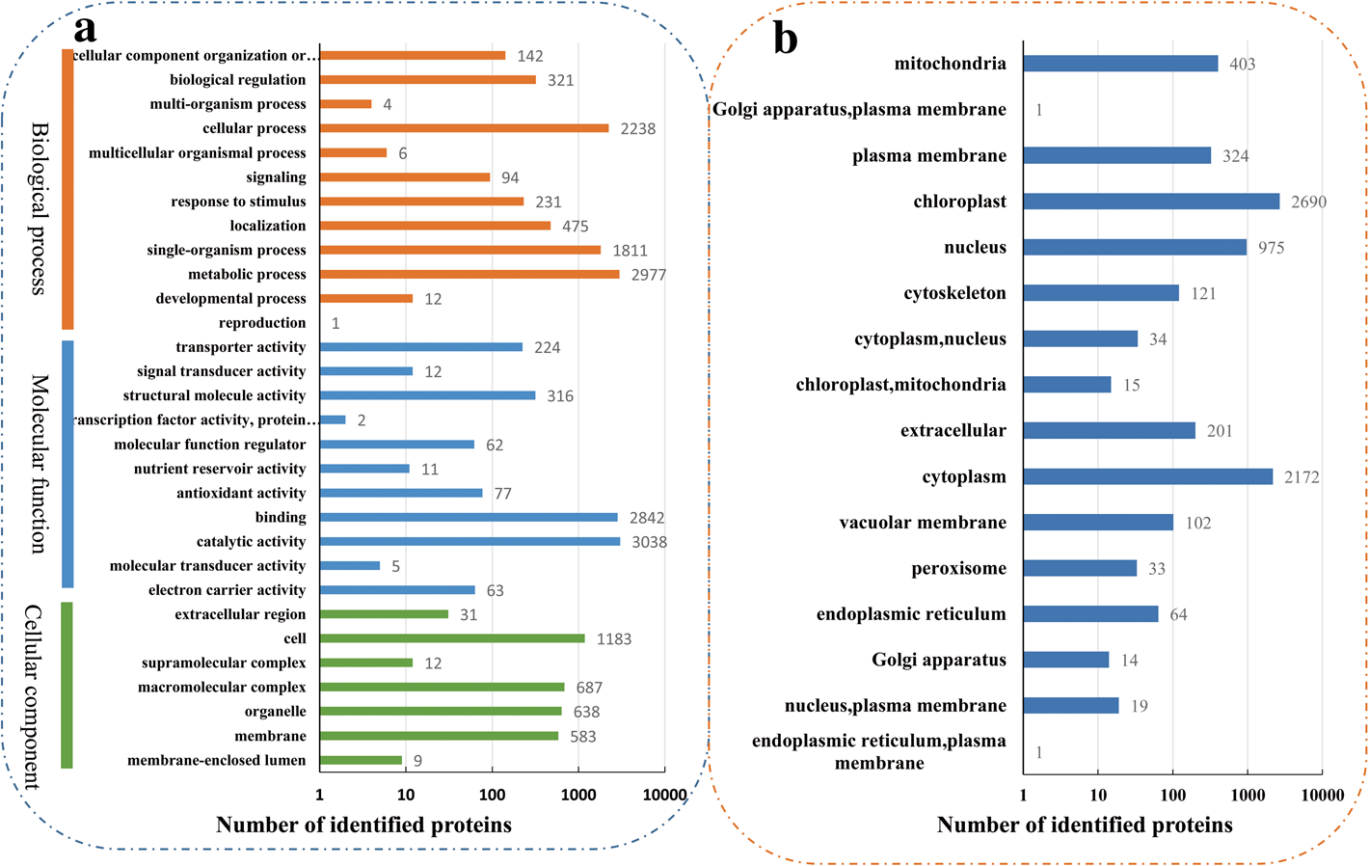
The information of all identified proteins. **a** GO analysis of all identified proteins. All proteins were classified by GO terms based on three categories: molecular function, biological process and cellular component. **b** Subcellular classify of identified proteins

After quality validation, 7179 proteins were detected, of which 5774 were quantified. The protein masses were distributed from 2.75 to 400 kDa (Fig. 1 c). Information on all identified proteins, including subcellular localization, Gene Ontology (GO) categories, KEGG pathways and domain descriptions, is presented in Additional file 4: Table S3. To further understand their functions, all identified proteins were annotated on the basis of GO terms based on three categories: cellular component, molecular function, and biological process (Fig. 2 a). In brief, ‘metabolic processes’ was the most commonly annotated category under the ‘biological process’ term, involving 2977 proteins, while 3038 proteins were annotated under ‘catalytic activity’ in the “molecular function” term. In the “cellular components” category, 1183 proteins were ‘cell’ -related proteins. Furthermore, all identified proteins were grouped according to their subcellular localization. A total of 16 subcellular locations were identified, including chloroplast (2690 proteins), cytoplasm (2172 proteins), and nucleus (975 proteins) (Fig. 2 b).

### Impacts of NaCl stress on the proteome levels of okra seedlings

**Fig. 3:**
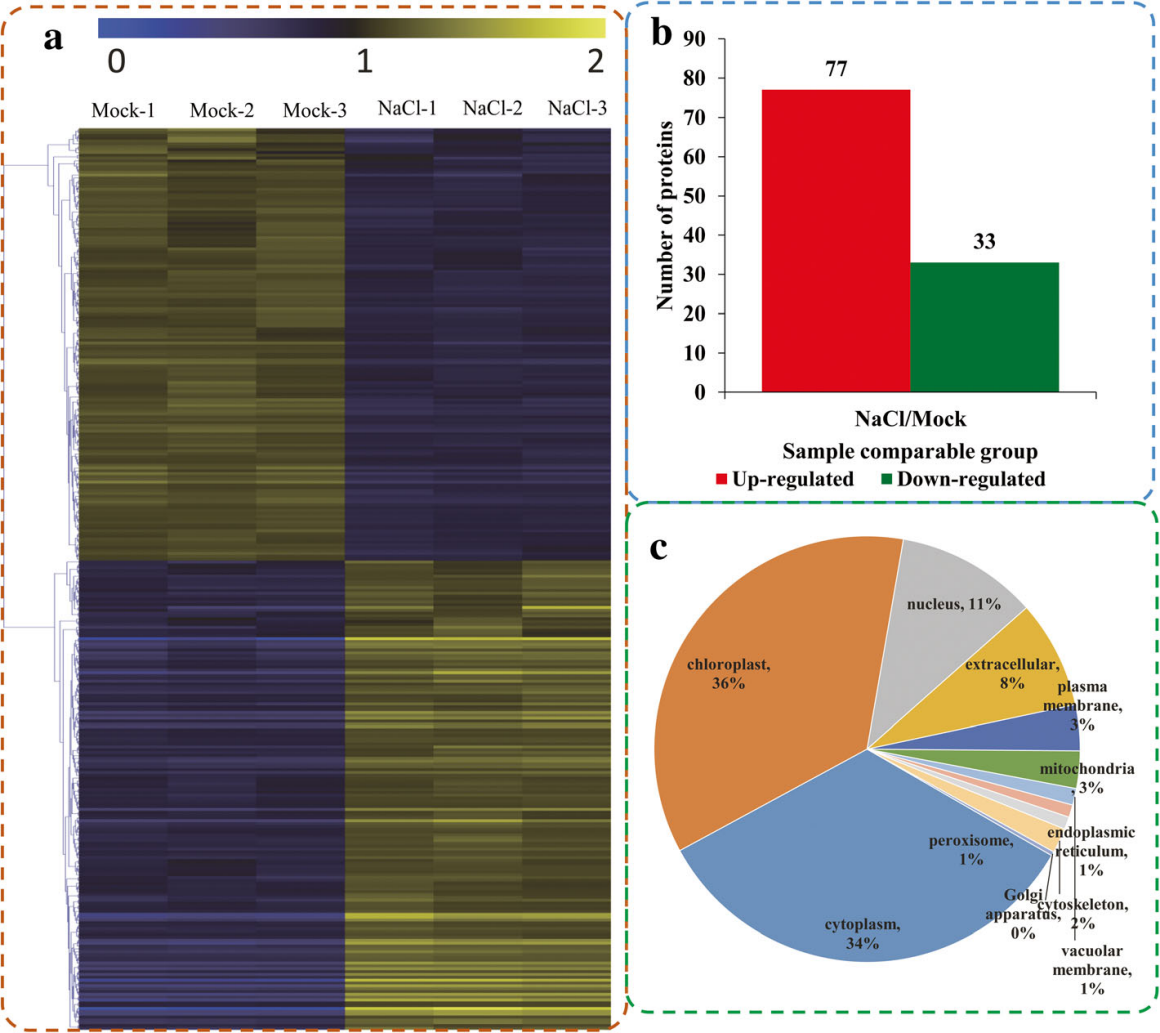
Impacts of salt stress treatment on proteome levels in okra. **a** Expression profiles of the DEPs response to salt stress. **b** The numbers of up- and down-regulated proteins in the salt treatment seedlings compared to the control seedlings. **c** Subcellular classify of DEPs

Among the quantifiable proteins, 317 were identified as DEPs between NaCl-treated and -untreated (control) seedlings based on the criteria: the ratios > 1.3 (up-regulated) and < 0.77 (down-regulated) coupled with *p* < 0.05 **(**Additional file 5: Table S4**).** The expression profiles of the DEPs in six samples were presented in a heatmap (Fig. 3 a). Of the DEPs, 165 proteins were upregulated and 152 proteins were down-regulated at 48 h after NaCl treatment compared with the control seedlings (Fig. 3 c). We also classified the DEPs according to their subcellular location (Fig. 3 d). A total of 11 subcellular components were represented, including chloroplast (113 proteins), cytoplasm (107 proteins) and nucleus (34 proteins).

Among the DEPs, the top five up-regulated proteins were a low molecular weight heat shock protein (Unigene58443_All, exhibiting a 9.196-fold increase), an uncharacterized protein (Unigene48512_All, 6.629-fold), two alpha-crystalline heat-shock-protein (Unigene98078_All 5.268 fold; Unigene89032_All 3.817 fold) and a heat shock protein (CL27466.Contig2_All, 3.668-fold). The top five down-regulated proteins were a ribonucleoprotein (CL26567.Contig9_All,0.492× control level), an At5g22580-like isoform of a stress-response A/B barrel domain-containing protein (CL21465.Contig3_All, 0.537), an unnamed protein (Unigene55082_All, 0.542), a conserved hypothetical protein (Unigene37040_All, 0.55), and a sucrose synthase Sus1 (Unigene2786_All, 0.553).

### Enrichment analysis of DEPs under NaCl treatment

For each of the DEPs identified, we performed enrichment analysis of GO, KEGG, and domain functions to determine whether each DEP had a significant enrichment trend with respect to certain functional types. The Fisher’s exact test was used to test the significance of the enrichment, and the *p*-value was transformed to the negative logarithm (−log_10_). The larger the p-value after transformation, the greater the enrichment of this function type.

**Fig. 4:**
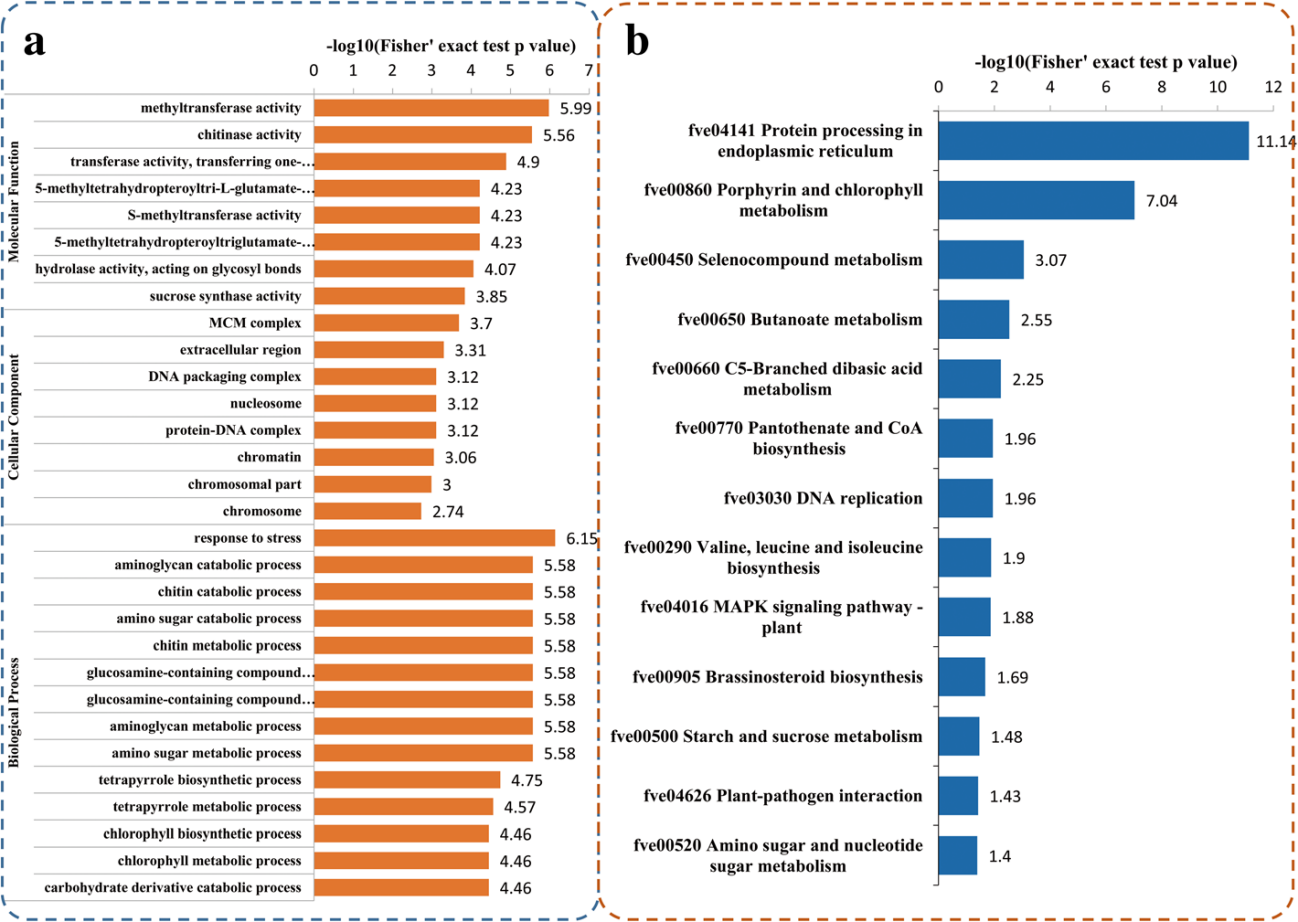
Enrichment analysis of the DEPs in okra after salt stress treatment. **a** Significantly enriched GO terms of the DEPs concerning molecular function, cellular component and biological process. **b** Significantly enriched KEGG terms of the DEPs

The significantly enriched molecular function GO terms were mainly associated with ‘methyltransferase activity’, ‘chitinase activity’, ‘transferase activity, transferring one-carbon groups’, ‘5-methyltetrahydropteroyltri-L-glutamate-dependent methyltransferase activity’, ‘S-methyltransferase activity’, ‘5-methyltetrahydropteroyltriglutamate-homocysteine S-methyltransferase activity’, ‘hydrolase activity, acting on glycosyl bonds’, and ‘sucrose synthase activity’. For the cellular component GO terms, the DEPs were significantly enriched with respect to ‘MCM complex’, ‘extracellular region’, ‘DNA packaging complex’, ‘nucleosome’, ‘protein-DNA complex’, ‘chromatin’, ‘chromosomal part’, and ‘chromosome’. The significantly enriched biological process GO terms were ‘response to stress’, ‘aminoglycan catabolic process’, ‘chitin catabolic process’, ‘amino sugar catabolic process’, ‘chitin metabolic process’, ‘glucosamine-containing compound metabolic process’, ‘glucosamine-containing compound catabolic process’, ‘aminoglycan metabolic process’, ‘amino sugar metabolic process’, ‘tetrapyrrole biosynthetic process’, ‘tetrapyrrole metabolic process’, ‘chlorophyll biosynthetic process’, ‘chlorophyll metabolic process’, and ‘carbohydrate derivative catabolic process’ (Fig. 4a). Our data annotated a number of DEPs as ‘response to stress’(Table 1).

**Table 1:** Identification of the DEPs involved in response to stress

Protein accession	Protein description	Organism	NaCl/mock Ratio	Regulated Type	*P* value
CL11615.Contig12_All	HSP90–1	*Glycine max*	1.309	Up	0.002683
CL1184.Contig9_All	catalase	*Gossypium hirsutum*	1.423	Up	4.87E-06
CL19789.Contig2_All	win2 recursor (gi|413,920,555|gb|AFW60487.1|)	*Zea mays*	1.405	Up	0.001196
CL23900.Contig3_All	class III peroxidase	Gossypium hirsutum	0.726	Down	0.00672
CL4062.Contig15_All	dehydrin	*Phaseolus vulgaris*	1.673	Up	0.009304
CL4797.Contig8_All	PREDICTED: prostaglandin G/H synthase 2-like	Glycine max	1.443	Up	0.002862
CL5745.Contig5_All	PR10–5-like protein	*Gossypium barbadense*	1.553	Up	0.000302
CL6768.Contig7_All	PREDICTED: chaperone protein ClpB3, chloroplastic-like	*Vitis vinifera*	1.571	Up	0.001656
Unigene22718_All	Hsp90	*Citrus sinensis*	1.355	Up	0.008159
Unigene4878_All	hypothetical protein SELMODRAFT (gi|300,155,731|gb|EFJ22362.1|)	Selaginella moellendorffii	2.549	Up	1.03E-06
Unigene5063_All	bacterial-induced peroxidase precursor	Gossypium hirsutum	1.38	Up	0.000245
Unigene79931_All	VDRG6(gi|83,356,301|gb|ABC16635.1|)	Gossypium hirsutum	1.421	Up	0.000262
Unigene88386_All	major latex-like protein	Gossypium hirsutum	0.731	Down	0.000142
Unigene77507_All	seed maturation protein PM37(gi|5,802,244|gb|AAD51625.1|)	Glycine max	1.378	Up	0.013424
CL11615.Contig14_All	hypothetical protein PRUPE_ppa002187mg	*Prunus persica*	1.878	Up	4.31E-06
CL1365.Contig2_All	hypothetical protein PRUPE_ppa009666mg	Prunus persica	0.589	Down	0.000177
CL16938.Contig3_All	uncharacterized protein (LOC100306513)	Glycine max	1.304	Up	2.98E-06
Unigene21193_All	hypothetical protein PRUPE_ppa010771mg	Prunus persica	1.354	Up	0.008936
Unigene62233_All	conserved hypothetical protein (gi|223,540,824|gb|EEF42384.1|)	*Ricinus communis*	1.321	Up	0.000725

**Fig. 5:**
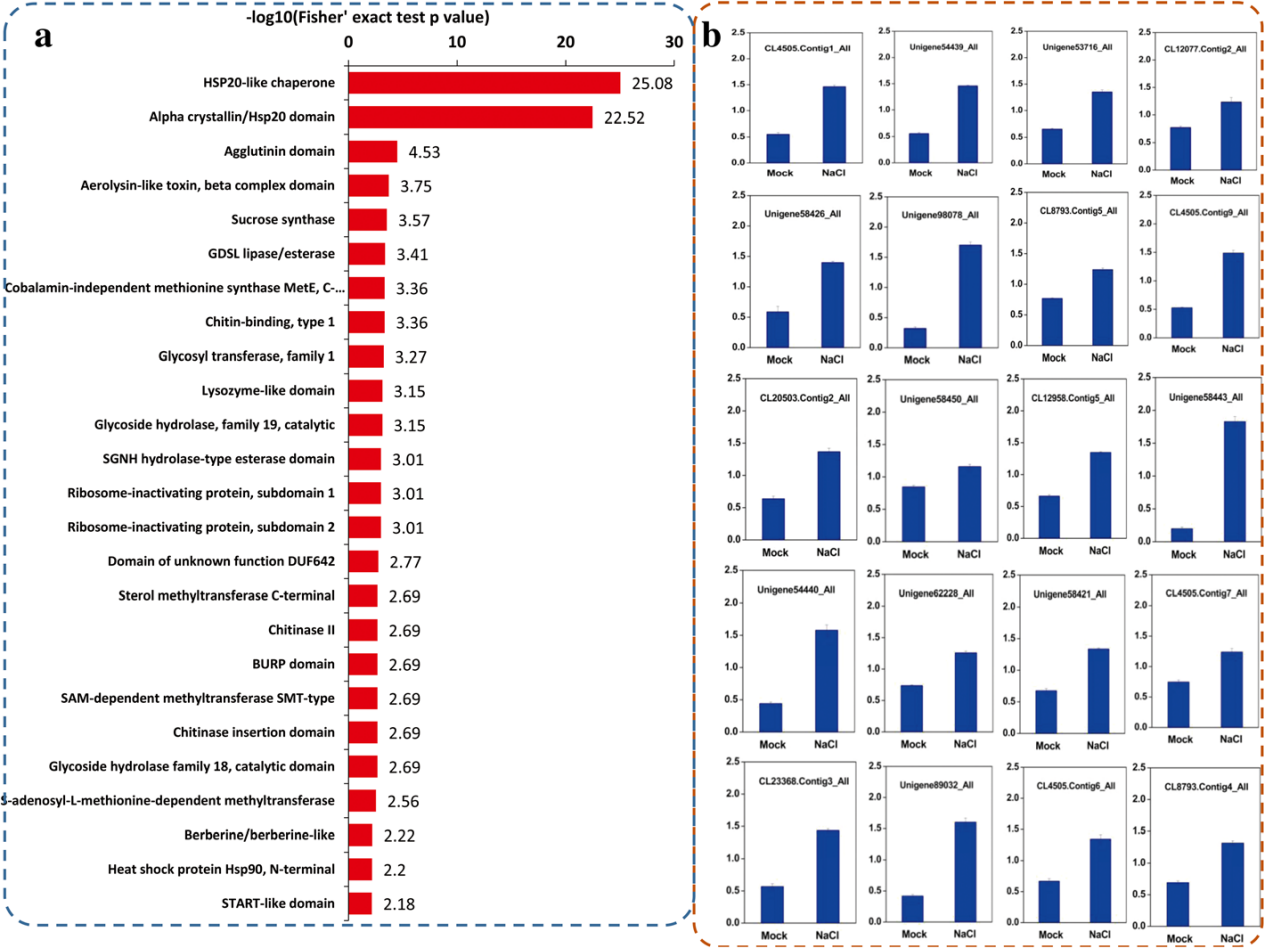
Protein domain enrichment analysis of the DEPs (**a**) and the accumulation of Hsp20 proteins (**b**) after salt stress treatment

KEGG enrichment analysis showed that DEPs were associated with 13 KEGG pathways. The three most significant pathways were ‘Protein processing in endoplasmic reticulum (fve04141)’, ‘Porphyrin and chlorophyll metabolism (fve00860)’, and ‘Selenocompound metabolism (fve00450)’ (Fig. 4 b). Protein domain enrichment analysis revealed that the DEPs were enriched with respect to 13 protein domains. The three most significant domains were ‘HSP20-like chaperone’, ‘Alpha crystallin’, and ‘Agglutinin domain’ (Fig. 5 a). Of the DEPs, 20 were HSP20-like chaperone proteins and their expressions are shown in Fig. 5 b.The expression levels of some HSP genes and ‘response to stress’related genes were basically consistent with the proteomic analyses (Additional file 6: Figure S2).

We categorized the DEPs into one of four groups (Q1 to Q4) on the basis of their differential expression multiple as follows: Q1 (0 < NaCl/control ratio < 1/1.5); Q2 (1/1.5 < ratio < 1/1.3), Q3 (1.3 < ratio < 1.5); and Q4 (ratio > 1.5) (Additional file 7 Figure S3). GO classification and KEGG enrichment were carried out for members of each Q group, and clustering analysis was carried out to detect any relationship between the functions of proteins and different expression multiples. The results revealed that the DEPs in Q1 (ratio < 1/1.5) were most strongly associated with ‘disaccharide metabolic process’, ‘oligosaccharide metabolic process’, ‘cellular polysaccharide metabolic’, ‘cellular glucan metabolic process’, and ‘glucan metabolic process’. The DEPs in Q4 (ratio > 1.5) were most strongly associated with ‘cell wall macromolecule metabolic process’, ‘cell wall macromolecule catabolic process’, ‘glucosamine-containing compound metabolic’, ‘glucosamine-containing compound catabolic’, ‘amino sugar catabolic process’, ‘chitin metabolic process’, and ‘aminoglycan catabolic process’ (Additional file 8 Figure S4). KEGG enrichment analysis revealed that the DEPs in Q1 were strongly associated with ‘amino-sugar and nucleotide-sugar metabolism’ and ‘starch and sucrose metabolism’, whereas the DEPs in Q4 were strongly associated with ‘brassinosteroid biosynthesis’, ‘endocytosis’, and ‘starch and sucrose metabolism ’ (Additional file 9 Figure S5).

### The identifcation of protein-protein interaction (PPI) networks among DEPs

**Fig. 6:**
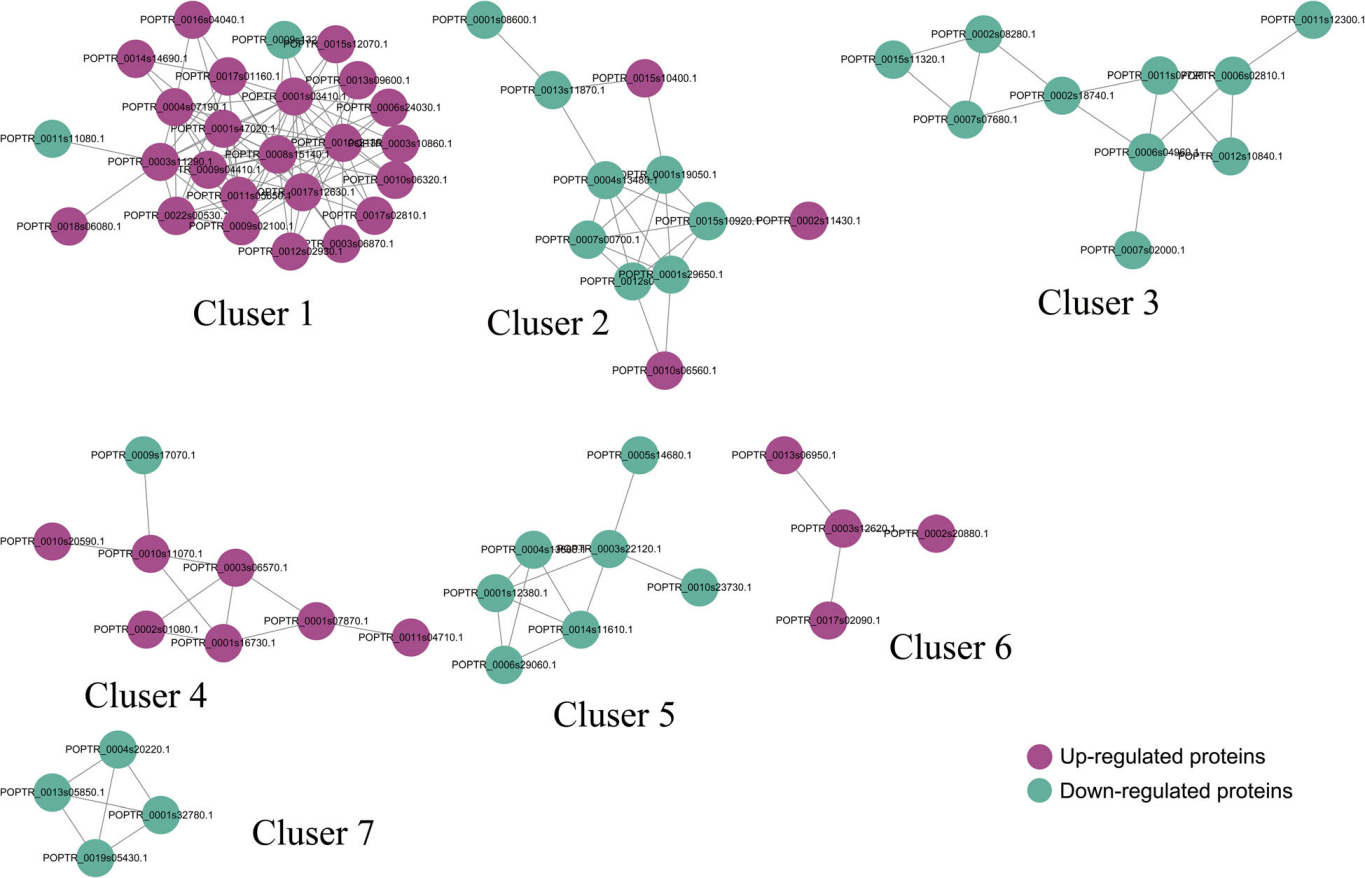
Interaction network of DEPs analyzed by Cytoscape sofware (version 3.0.1) The upregulated and downregulated proteins in the clusters were shown in cyan and green, respectively. Detailed information on node and proteins can be found in Additional file 10 Table S5 and Additional file 11 Table S6.

The identification of PPI networks through bioinformatics analysis is considered to be a useful tool for formulating testable hypotheses to determine the unknown protein functions [29].To further understand the protein regulatory network of okra in respose to salt-stress, a PPI map among the DEPs was generated by cytoscape software. A total of 69 DEPs, including 37 up and 32 downregulated peptides, were shown in the PPI network (Fig. 6). The detailed node and network information were listed in Additional file 10 Table S5 and Additional File 11 Table S6. Seven enriched interaction clusters were identified from the data analysis. Cluster 1 consisted of 16 ‘protein processing in endoplasmic reticulum’ related proteins and ‘10 binding’ related proteins. Cluster 2 consisted of six ‘Carbon metabolism’ related proteins, two ‘C5-Branched dibasic acid metabolism’ proteins, a P-loop containing nucleoside triphosphate hydrolase protein, a branched-chain amino acid aminotransferase protein and a Hydroxymethylglutaryl-coenzyme A synthase protein. Eight Porphyrin and chlorophyll metabolism related proteins have been identified in cluster 3. For cluster 4, several enzymes, such as primary-amine oxidase, delta-1-pyrroline-5-carboxylate synthetase,glutamyl-tRNA synthetase, catalase polyphenol oxidase and glutamate decarboxylase, have been included. For cluster 5, four ‘organic cyclic compound binding’ proteins, two ‘DNA binding’proteins and a pyruvate dehydrogenase E1 component beta subunit protein, have been identified. Three glutathione S-transferase proteins and a glutathione peroxidase protein were identifed in cluster 6. Three 5-methylt etrahydropteroyltriglutamate--homocysteine methyltransferase proteins and an adenosylhomocysteinase protein were identified in cluster 7.

## Discussion

Soil salinity is one of the main abiotic stresses limiting plant growth and agricultural productivity. Understanding the mechanisms that protect plants from salt stress will help in the development of salt-stress-tolerant crop and vegetable cultivars. Okra is now widely cultivated all over the world [30]. Due to the complex allopolyploid genome of okra (probably, 2n = 130–140), little attention has been paid to the genetic improvement of this crop until recently [31]. In the present work, a TMT-based proteomic technique was employed to analyze the proteins differentially expressed between the control and NaCl-treated seedlings over the first 48 h. These results will enhance our understanding of the regulatory mechanisms involved in okra response to salt stress.

High-throughput proteomic analysis has been used to reveal the responses of plants to salt stress at the protein level. Using the 2-DE and MALDI-TOF-MS method, 34 salt-stress-responsive-protein spots in NaCl-treated cucumber roots were successfully identified by Du et al. and 53 protein spots were significantly regulated by NaCl, as identified by Yuan et al. [24, 32]. A total of 128 DEPs was identified in the roots of NaCl-treated upland cotton roots using the iTRAQ-based proteomic technique [26]. In our study, 7179 proteins and 317 DEPs were identified, which was far more than the protein numbers reported by the previous studies. The large number of identified proteins gives us the opportunity to conduct a more in-depth and comprehensive analysis of proteins responsive to salt stress than that has been achieved by other studies.

Salt stress leads to the accumulation of reactive oxygen species (ROS), which oxidize cellular components (proteins, lipids, carbohydrates and DNA), irreversible damage to plant cells [33, 34]. In plants, ROS can be scavenged by catalases (CAT), peroxidases (POD), ascorbate peroxidases (APX), glutathione S-transferases (GST) and superoxide dismutases (SOD). In the present study, a POD, a POD precursor, and a CAT protein were identified. The expression of these proteins suggested that salt stress induced changes in the antioxidant defense system of okra seedlings. In addition to redox-related proteins, plants have evolved a stress cross-tolerance mechanism that adapts to different stresses [35]. From our TMT data, a biotic-stress-related proteins, pathogenesis-related protein class 10 (PR10–5, CL5745.Contig5_All), which mediates resistance to pathogen attack (Coumans et al., 2009), was induced under salt-stress conditions. A major latex protein (MLP) was down regulated in okra shoots under salt stress, which was consistent with the results from soybean leaf proteomics in response to salt stress [36]. The MLP subfamily is known to be involved in fruit and flower development and in various stress responses [36, 37]. However, whether MLPs levels are associated with enhanced salt tolerance in plants is far from being clear, though they might represent a novel salt-stress-responsive protein in plants [36]. Furthermore, a drought-stress-related protein, dehydrin, also responded to salt stress in this study. These proteins provide new insights into the cross-tolerance mechanisms in okra seedlings to biotic and abiotic stresses.

Under salt stress, the cytoskeleton is rapidly remodeled to allow cell size adjustment to maintain normal cell swelling pressure [35]. In salt-treated shoots of okra seedlings, we found that three DEPs were involoved in “cell wall macromolecule catabolic/ process” which might affect cell wall remodeling. Our results indicated that a large number of DEPs were associated with various important biological processes such as salt signaling and multiple metabolism. The chlorophyll content decreased significantly in cotton under salt stress [38], and it has been reported that a reduction in photosynthetic protein accumulation occurs in chlorophyll biosynthesis mutants [39]. There were 13 DEPs associated with ‘Porphyrin and chlorophyll metabolism’ in the present study. It is reported that the mitogen-activated protein kinase (MAPK) family plays a variety of roles in plant intracellular and extracellular signal transduction by transmitting information from sensors to responders, and the MAPK family acts as a convergence point in abiotic stress signal transduction [40]. Both biotic and abiotic stresses cause protein misfolding or the accumulation of unfolded proteins, which can be sensed by specific receptor proteins on the endoplasmic reticulum membrane, casuing endoplasmic reticulum stress [41]. Proper protein folding is important for normal cell function under salt stress [26], and daptation to salt stress requires complex metabolic rearrangements and interactions among multiple metabolic pathways [36].

Heat-shock proteins (HSPs) can be induced by a range of stresses in almost all organisms, and their concentrations can increase rapidly in plants in response to adverse environmental conditions [42–45]. HSPs is a class of evolutionarily conserved proteins, and can be divided into five families, namely HSP100, HSP90, HSP70, HSP60, and HSP20, based on molecular weight and sequence homology [46, 47]. There is growing evidence that HSPs are closely associated with salt stress tolerance. The PfHSP17.2-overexpressing transgenic *Arabidopsis* was more tolerant to heat and salt than were the wild-type plants, while transformation with a rose cytosolic class I small HSP (sHSP), RcHSP17.8, conferred increased tolerance to salt stress in *Arabidopsis* [48, 49]. An *Arabidopsis* cytosolic class II sHSP, AtHSP17.6A, was induced by osmotic stress, while a *Populus trichocarpa HSP, pthsp*17.8, was involved in enhancing tolerance to heat and salt stresses [50, 51]. Among the salt-stress-induced DEPs in okra, a number of HSPs were identified, and 20 HSP20 proteins were significantly upregulated by salt stress, suggesting molecular cross-talk between heat shock responses and salt stress.

## Conclusions

In the present study, a TMT-based proteomics technique was used to investigate the DEPs induced after exposure of okra seedlings to salt or water for 48 h. In total, 317 DEPs were identified, 165 of which displayed upregulation and 152 downregulation under salt-stress conditions. We obtained new information on okra seedling proteins and their roles in salt-stress response. A number of DEPs were mainly involved in the biological processes of response to stress and metabolism. The diversity of the proteins affected by salt stress indicates that the metabolism of okra seedlings has obvious flexibility, which may contribute to the survival of okra under salt stress. Our findings provide fundamental resources for identifying candidate proteins and molecular mechanisms involved in the response of okra plants to salt stress.

## Methods

### Plant materials

Seeds of the okra cultivars ‘Wufu’ were obtained from the Vegetable Research Institute of Zhejiang Academy of Agricultural Sciences, Hangzhou, China. The seeds were disinfected with 10% sodium hypochlorite for 10 min, and then washed three times with distilled water. Seeds were sown in plastic trays containing peaty soil. From 1 week after germination, half-strength Hoagland’s nutrient solution was applied to the trays every 3 days. Seedlings were grown in an artificial illumination incubator with 24/28 °C, with a light intensity of 300 μmol m^− 2^ s^− 1^ a photoperiod of 12-h light/12-h dark, and a relative humidity of 60%. Two week after germination, seedlings of uniform size were transferred to flowerpots with 7 × 7 × 10 cm (length ×width× height) size with one seedling transplanted to each pot. Three weeks after germination, 20 ml water (control) or of 300 mmol L^− 1^ NaCl (salt stress) were applied to each pot. After 48 h of treatment (control or salt stress), the above-ground part of seedlings was used to extract protein.

### Protein extraction

The appropriate amount of seedling tissue was snap-frozen and ground in liquid nitrogen, at which point the powder was transferred to a 5 mL centrifuge tube. A four-fold volume of the lysis buffer (containing 10 mM dithiothreitol, 1% protease inhibitor Cocktail (P8849, Sigma-Aldrich, Beijing, China) and 2 mM EDTA) was added to each sample and sonicated three times on ice, using a high-intensity ultrasonic processor (JY99-IIDN,Scientz, Ningbo, China). An equal volume of Tris-saturated phenol (pH 8.0) was added, and the mixture was vortexed for 5 min. After centrifugation (4 °C, 10 min, 5000×g), the upper phenol phase was removed and transferred to a clean centrifuge tube. Proteins were precipitated by addition of five volumes of 0.1 M ammonium sulfate-saturated methanol and incubation at − 20 °C for overnight. After centrifugation at 4 °C for 10 min, the supernatant was discarded, and the pellet was washed once with ice-cold methanol, followed by washing three times with ice-cold acetone. The protein was re-dissolved in 8 M urea and the protein concentration was determined with the bicinchoninic acid BCA kit (P0012, Beyotime, Shanghai, China), according to the manufacturer’s instructions.

For trypsin digestion, the protein solution was reduced with 5 mM dithiothreitol for 30 min at 56 °C and alkylated with 11 mM iodoacetamide for 15 min at room temperature in darkness. The protein sample was then diluted by adding 100 mM TEAB (triethylammonium bicarbonate) to achieve a urea concentration of less than 2 M. Finally, trypsin was added at 1:50 (trypsin:protein) mass ratio for the first digestion overnight and 1:100 (trypsin:protein) mass ratio for a second 4 h-digestion.

### Tandem mass tag (TMT) labeling, HPLC fractionation, and LC-MS/MS analysis

After trypsin digestion, the peptide was desalted by solid-phase extraction (SPE) on a Strata X C18 column (Phenomenex, Torrance, CA, USA) and dried by vacuum centrifugation. Peptides were reconstituted in 0.5 M TEAB and processed according to the operating instruction for the TMT kit (Thermo Fisher Scientific, Shanghai, China). Briefly, one unit of TMT reagent was thawed and reconstituted in acetonitrile. The peptide mixtures were then incubated for 2 h at room temperature and pooled, desalted and dried by vacuum centrifugation.

The tryptic peptides were fractionated into fractions by high pH reverse-phase high-performance liquid chromatography (HPLC) using Agilent 300 Extend C18 column (5 μm particle size, 4.6 mm internal diameter× 250 mm length) (Agilent, Shanghai, China). Briefly, peptides were first separated with a linear gradient of 8 to 32% acetonitrile (pH 9.0) over 60 min into 60 fractions. Then, the peptides were combined into 18 fractions and dried by vacuum centrifugation.

The tryptic peptides were dissolved in 0.1% (v/v) formic acid buffer (solvent A), directly loaded onto a home-made reversed-phase analytical column (15 cm length× 75 μm internal diameter). The gradient consisted of an increase from 6 to 23% solvent B (0.1% formic acid in 98% acetonitrile) over 26 min, 23 to 35% in 8 min and climbing to 80% in 3 min then holding at 80% for the last 3 min, all at a constant flow rate of 400 nL/min on an EASY-nLC 1000 Ultra-performance liquid chromatography (UPLC) system (Thermo Fisher Scientific, Waltham, MA, USA). The peptides were subjected to a nitrogen solution index (NSI) source followed by tandem mass spectrometry (MS/MS) in a Q Exactive™ Plus mass spectrometer (Thermo Fisher Scientific, Shanghai, China) coupled online to the UPLC. The electrospray voltage applied was 2.0 kV. The m/z scan range was 350 to 1800 for full scan, and intact peptides were detected in the Orbitrap at a resolution of 70,000. Peptides were then selected for MS/MS using a normalized collision energy (NCE) setting at 28 and the fragments were detected in the Orbitrap at a resolution of 17,500. Automatic gain control (AGC) was set at 5E4. Fixed first mass was set at 100 m/z.

### Database search and TMT quantification

The resulting MS/MS data were used to searched against a published okra transcriptome data up-loaded by our lab [31] (NCBI Sequence Read Archive database accession: SRP130180) using the MaxQuant search engine (v.1.5.2.8) concatenated with the reverse decoy database. The enzyme digestion mode was set to Trypsin/P, allowing for up to two missing cleavages. The mass tolerance for precursor ions was set at 20 ppm in the first search and at 5 ppm in the main search, and the mass tolerance for fragment ions was set at 0.02 Da. Carbamidomethyl-modified cysteine residues were specified as a fixed modification, and oxidation of methionine was specified as a variable modification. The quantitative method is set to TMT-6plex. False discovery rate was adjusted to low than 1% and minimum score for peptides was set at greater than 40. For TMT quantification, the ratios of the TMT reporter ion intensities in MS/MS spectra (m/z 126–131) from raw data sets were used to calculate fold changes between samples. For each sample, the quantification was mean-normalized at peptide level to center the distribution of quantitative values. Protein quantitation was then calculated as the median ratio of corresponding unique or razor peptides for a given protein.

### Annotation methods and functional enrichment

Gene Ontology (GO) annotation of the proteome was derived from the UniProt-GOA database (http://www.ebi.ac.uk/GOA). Firstly, identified protein IDs were converted to UniProt ID and then mapped to GO IDs by the protein ID. The InterProScan software was used to annotated each protein’s GO functional on the basis of protein sequence alignment method if these proteins had not been annotated by the UniProt-GOA database. Then each protein was classified on the basis of three categories: molecular function, biological process and cellular component. For each category, a two-tailed Fisher’s exact test was employed to test the significance of the enrichment of each differentially expressed protein (DEP) against all identified proteins. Any GO with a corrected *p*-value < 0.05 was considered to be significant.

The Kyoto Encyclopedia of Genes and Genomes (KEGG) database was used to annotate protein pathways, and KAAS (KEGG Automatic Annotation Server, https://www.genome.jp/tools/kaas/) was used to annotate each protein’s KEGG database description. The annotation result was mapped on to the KEGG pathway database using KEGG the mapper (https://www.genome.jp/kegg/mapper.html). The interPro domain database (http://www.ebi.ac.uk/interpro/) was used to analyse functional descriptions of identified proteins domains. The KEGG database was used to identify enriched pathways using a two-tailed Fisher’s exact test to test the significance of enrichment of each differentially expressed protein against all identified proteins. A pathway with a corrected *p*-value < 0.05 was considered to be significant. These pathways were classified into hierarchical categories as described on the KEGG website.

Wolfpsort (a subcellular localization prediction software, PSORT/PSORT II version) was used to predict subcellular localization. The InterPro database was researched and a two-tailed Fisher’s exact test was employed to test the significance of the enrichment of each DEP against all identified proteins. Protein domains with a p-value < 0.05 were considered to be significantly different.

For further hierarchical clustering based on different protein functional classification (such as: GO term, protein domain and KEGG pathway enrichment), we first collated all the categories obtained after enrichment along with their *p*-values, and then filtered for those categories which were at least enriched in one of the clusters with a p-value < 0.05. Each filtered p-value matrix was transformed by the function x = −log_10_ (p-value). Finally these x values were z-transformed for each functional category. These z scores were then clustered by one-way hierarchical clustering (Euclidean distance, average linkage clustering) in Genesis. Cluster membership were visualized by a heat map using the “heatmap.2” function from the “gplots” R-package.

### Protein-protein interaction network

All differential expression protein name identifiers were searched against the STRING database version 10.5 for protein-protein interactions. Only interactions between the proteins belonging to the searched data set were selected, thereby excluding external candidates. STRING defines a metric called “confidence score” to define interaction confidence; we fetched all interactions that had a confidence score ≥ 0.7 (high confidence). Interaction network form STRING was visualized in Cytoscape.

### Quantitative real-time PCR validation

Total RNA was extracted using a RNAiso for Polysaccharide-rich Plant Tissue Kit according to the manufacturer’s protocol (Code:9752, TAKARA, Beijing, China). First-strand cDNA synthesis was carried out using a PrimeScript™RT Master Mix (Perfect Real Time) according to the manufacturer’s protocol (Code:RR036, TAKARA, Beijing, China). QRT-PCR was performed using TB Green Premix Ex Taq II (Tli RNaseH Plus) Kit (Code:RR820, TaKaRa, Dalian, China) and an LightCycler480 instrument (Roche, Basel, Switzerland). The primer sequences were listed in Additional file 1: Table S1. The *AeACTIN* (*CL25873.Contig1_All*) was used as an internal standard to calculate relative fold-differences based on comparative cycle threshold (2^−ΔΔCt^) values.

## Additional files

Additional file 1: **Table S1.** The primer sequences for qRT-PCR. (XLSX 10 kb)

Additional file 2: **Figure S1.** Pearson’s correlation of proteomes from different sample groups. Protein from each group were extracted in three biological replicates. Proteins were trypsin digested and then analyzed by HPLC-MS/MS. (TIF 417 kb)

Additional file 3: **Table S2.** The detail information of identified peptides pertinent to detected proteins. (XLSX 5698 kb)

Additional file 4: **Table S3.** The detail information of all identified peptides. (XLSX 1618 kb)

Additional file 5: **Table S4.** The detail information of DEPs. (XLSX 91 kb)

Additional file 6: **Figure S2.** Real-time quantitative PCR validation of several selected Salt responsive genes. The data were analyzed by three independent repeats, and standard deviations were shown with error bars. Significant differences in expression level were indicated by “*”. (TIF 828 kb)

Additional file 7: **Figure S3.** Comparable group of the DEPs according to their quantification ratios. (TIF 2048 kb)

Additional file 8: **Figure S4.** The heat map of cluster analysis based on enriched ‘Biological Proces’ GO term. (TIF 1564 kb)

Additional file 9: **Figure S5.** The heat map of cluster analysis based on enriched KEGG Pathways. (TIF 1328 kb)

Additional file 10: **Table S5.** Node Information of PPI. (XLSX 26 kb)

Additional file 11: **Table S6.** Network Information of PPI. (XLSX 23 kb)

## Abbreviations

DEP: Differentially expressed protein
FDR: False discovery rate
GO: Gene Ontology
iTRAQ: isobaric tag for relative and absolute quantitation
KEGG: Kyoto Encyclopedia of Genes and Genomes
LC-MS/MS: Liquid chromatography electrospray ionization tandem mass spectrometry
TMT: Tandem Mass Tag
