# Supplementary figures and images for: Comparative proteomic analysis of okra (Abelmoschus esculentus L.) seedlings under salt stress
Source: BMC Genomics. 2019 May 16;20:381. doi: 10.1186/s12864-019-5737-7 (PMC6521433; doi:10.1186/s12864-019-5737-7)

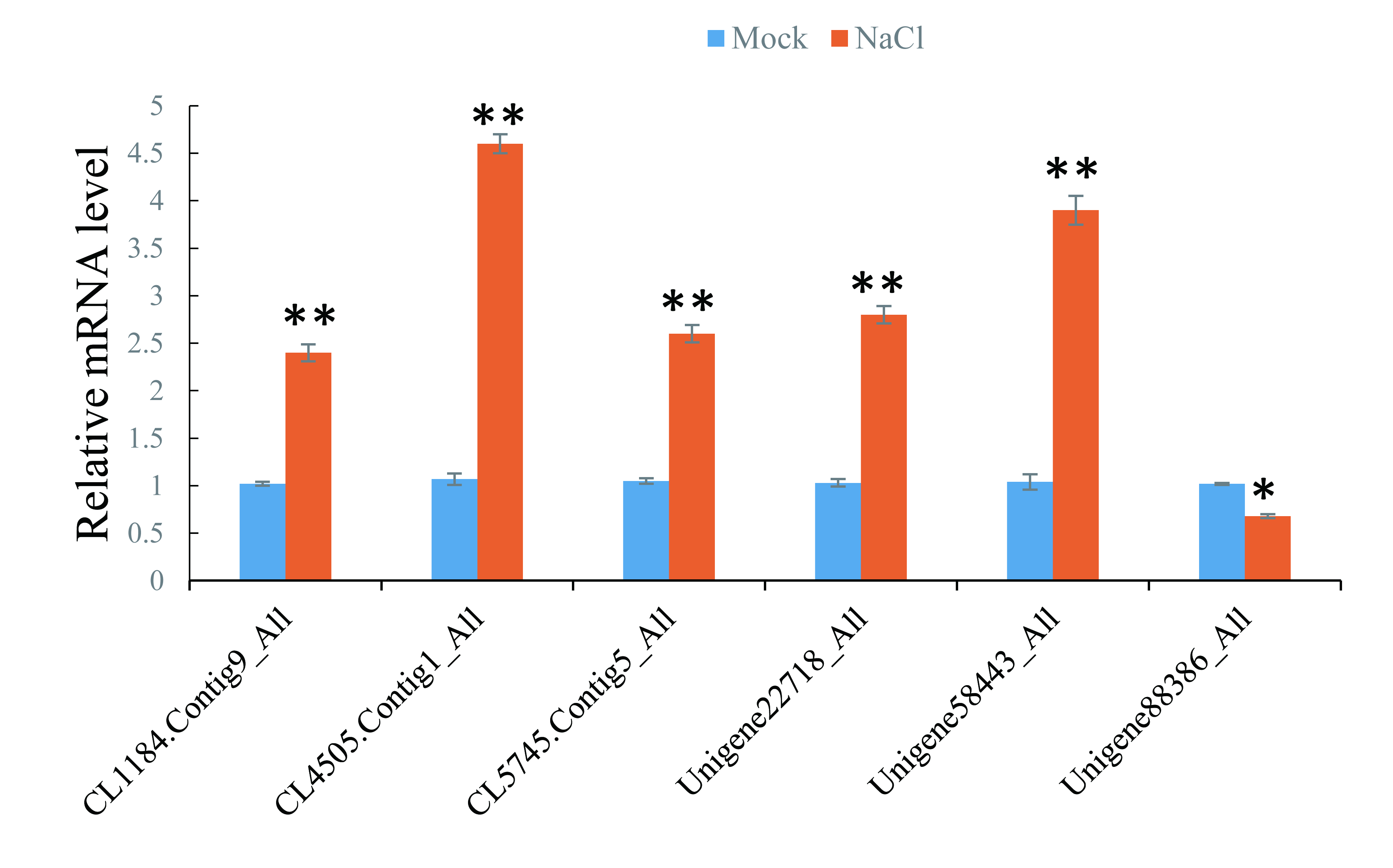

Supplement: Supplementary file 2 — Figure S1. Pearson’s correlation of proteomes from different sample groups. Protein from each group were extracted in three biological replicates. Proteins were trypsin digested and then analyzed by HPLC-MS/MS. (TIF 417 kb) [file 12864_2019_5737_MOESM2_ESM.tif]

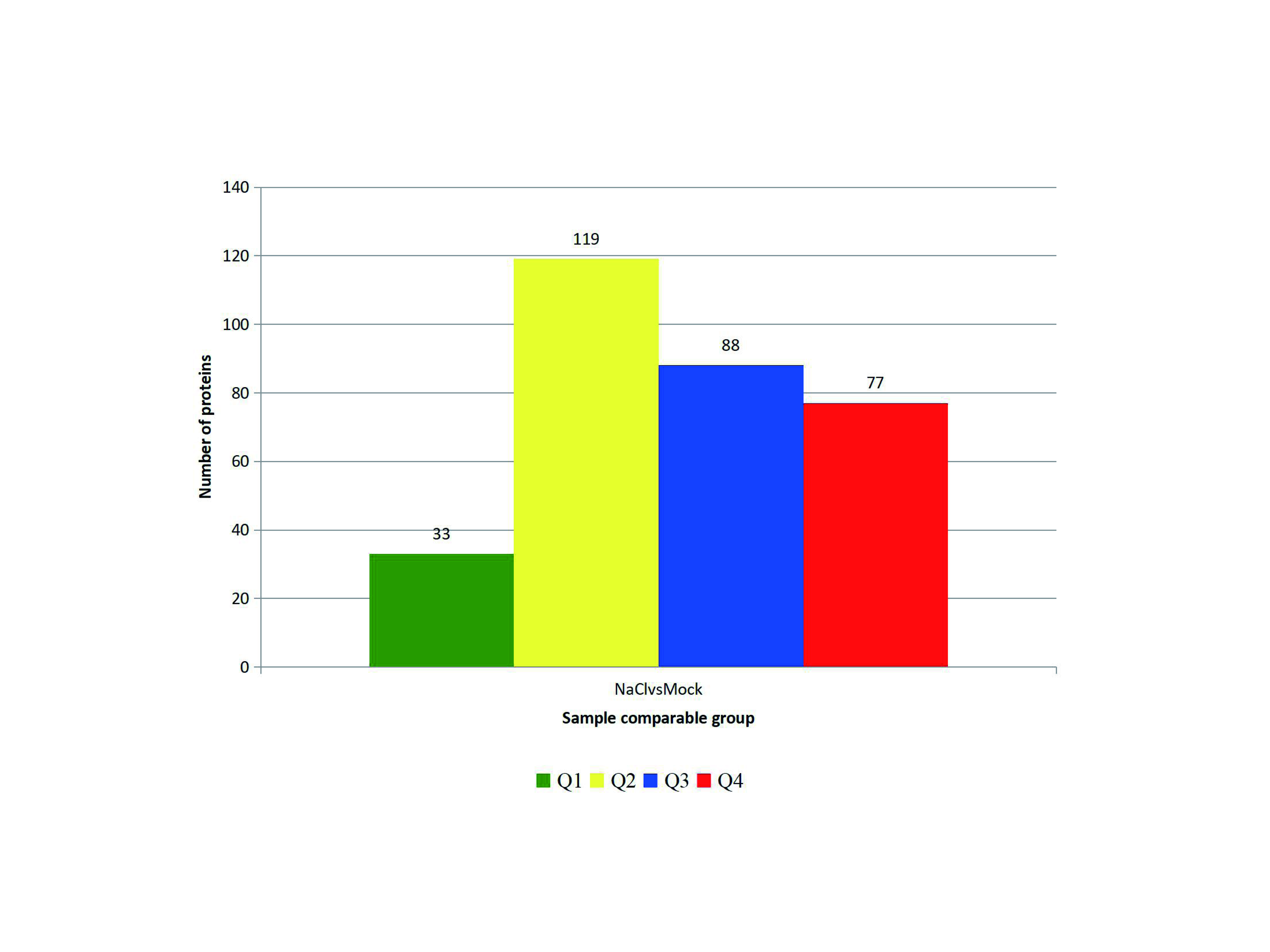

Supplement: Supplementary file 7 — Figure S3. Comparable group of the DEPs according to their quantification ratios. (TIF 2048 kb) [file 12864_2019_5737_MOESM7_ESM.tif]

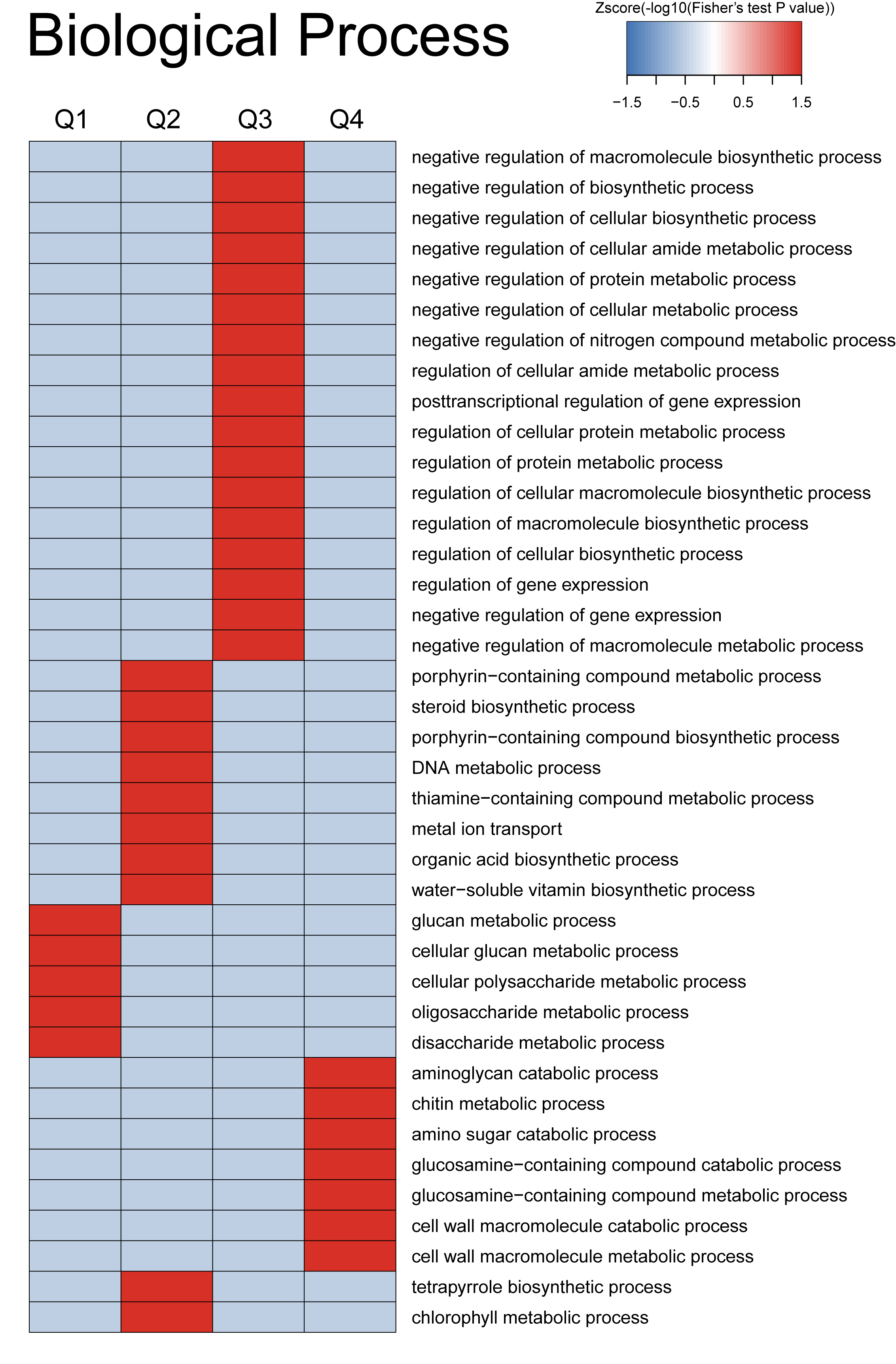

Supplement: Supplementary file 8 — Figure S4. The heat map of cluster analysis based on enriched ‘Biological Proces’ GO term. (TIF 1564 kb) [file 12864_2019_5737_MOESM8_ESM.tif]

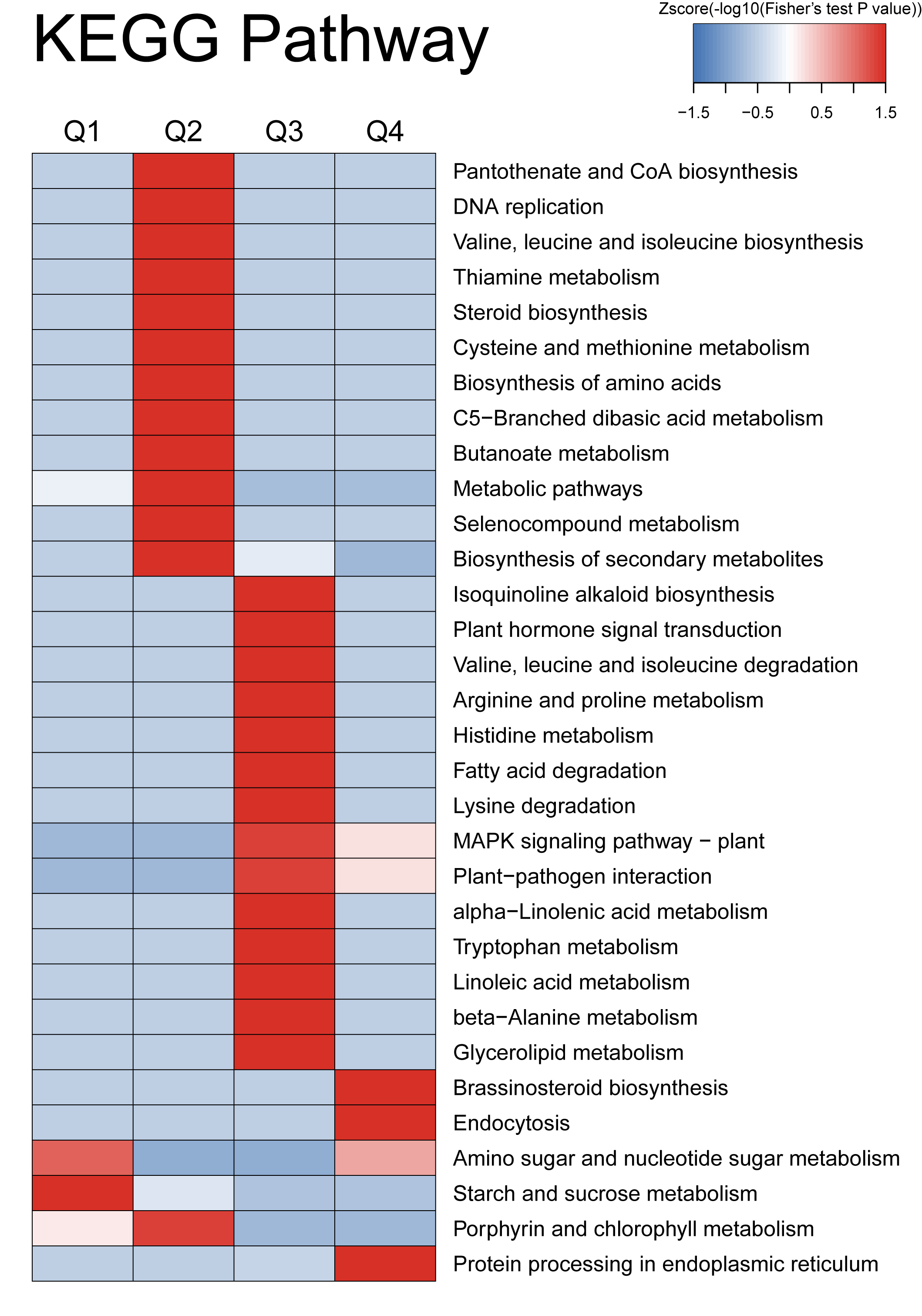

Supplement: Supplementary file 9 — Figure S5. The heat map of cluster analysis based on enriched KEGG Pathways. (TIF 1328 kb) [file 12864_2019_5737_MOESM9_ESM.tif]
